# Case Report: Recurrent colonic metastasis from lung cancer—diagnostic pitfalls and therapeutic challenge of a peculiar case

**DOI:** 10.3389/fsurg.2023.1288940

**Published:** 2023-12-22

**Authors:** Salvatore Tramontano, Gerardo Sarno, Vera Prisco, Anna Mirea Tedesco, Antonio Gargiulo, Umberto Bracale

**Affiliations:** ^1^ Department of Physics, University of Salerno, Salerno, Italy; ^2^ Department of General and Emergency Surgery, Ospedali Riuniti San Giovanni di Dio e Ruggi d'Aragona, Salerno, Italy; ^3^ Department of Surgery, University of Naples Federico II, Naples, Italy

**Keywords:** colonic metastasis, lung cancer, relapse, colon, lung

## Abstract

Lung cancer (LC) mortality exceeds 20%, and detecting metastases from LC is becoming a challenging step in understanding the real prognostic role of specific localization. We report a case of a patient with lung metastasis to the colon with local recurrence at the anastomosis after radical resection for metastasis. In both cases, the diagnosis was on oncological follow-up, and surgery was offered in consideration of reasonable life expectancy, good control of LC, and high risk of intestinal occlusion. A 67-year-old male, with a history of LC 18 months ago, was referred to our surgical unit after a positron emission tomography CT total body, where an area of intense glucose metabolism (SUV max: 35.6) at the hepatic colic flexure was reported. A colonoscopy revealed an ulcerated, bleeding large neoplasm distally to hepatic flexure, almost causing resulting total occlusion. Histologic examination revealed a tumor with complete wall thickness infiltration, which appears extensively ulcerated, from poorly differentiated squamous carcinoma (G3), not keratinizing, with growth in large solid nests, often centered by central necrosis. Two of the 30 isolated lymph nodes were metastatic. The omental flap and resection margins were free from infiltration. The malignant cells exhibited strong positive immunoreactivity only for p40. The features supported metastatic squamous carcinoma of lung origin rather than primary colorectal adenocarcinoma. After 8 months from surgery, intense Fluorodeoxyglucose (FDG) uptake of tissue was confirmed in the transverse colon. Colonoscopy evidenced an ulcerated substenotic area that involved ileocolic anastomosis on both sides. Reoperation consisted of radical resection of ileocolic anastomosis with local lymphadenectomy and ileotransverse anastomosis. The second histologic examination also revealed poorly differentiated squamous carcinoma (G3), not keratinizing, with positive immunoreactivity only for p40, suggesting the origin of LC. This case report confirmed that the possibility of colonic secondary disease should be part of the differential diagnosis in asymptomatic patients and those with a history of LC diagnosis. In addition, relapse of colonic metastasis is infrequent but should be considered during follow-up of LC. More studies on colonic metastasis of LC are required to better understand the clinical features and outcomes.

## Introduction

Nowadays, lung cancer (LC) mortality exceeds 20% (1), and detecting metastases from LC is becoming a challenging step in understanding the real prognostic role of specific localization (2). A new concept of overall survival (OS) in pulmonary metastatic disease was recently discussed (3). Non-small cell lung carcinoma (NSCLC) accounts for the majority of all LC cases (1). A metastatic disease often occurs at the time of diagnosis, regardless of the primary LC type. The most common sites of metastasis are the brain (47%), bone (36%), liver (22%), adrenal glands (15%), thoracic cavity (11%), and distant lymph nodes, but numerous locations were also described in the literature (2, 4). Colonic metastases are rare. Large bowel metastases have an incidence rate of approximately 12% in autoptic series (4). The report estimated lower incidence, mostly because symptomatic colonic metastasis infrequently occurs. Colonic metastases are significantly rare, with less than 50 cases of colonic metastasis from an LC reported to date (5). Clinicians should have a high index of suspicion and a low threshold for intestinal tract investigation when primary LC patients present with abdominal symptoms (6). We report a case of lung metastasis to the colon with local recurrence at the anastomosis after radical resection for metastasis. In both cases, the diagnosis was on oncological follow-up, and surgery was offered in consideration of reasonable life expectancy, good control of LC, and high risk of intestinal occlusion. No report was described in the literature of double colonic metastasis with relapse at the same location of colonic metastasis, although radical resection was performed and documented by histopathology.

## Case description: diagnostic assessment

A 67-year-old male was referred to our surgical unit by his oncologist after a positron emission tomography CT (PET-CT) total body, where an area of intense glucose metabolism (SUV max: 35.6) at the hepatic colic flexure was reported. LC consisted of a squamous type non-small cell cancer (S-NSCLC) and was diagnosed 18 months ago, with a local advanced pattern (T2N3M0). The patient was treated with immunotherapy, according to the international guidelines. He was thought to be in remission following 18 months of pembrolizumab. This pattern of disease was stable until December 2021, when PET-CT documented colonic metastasis. This was confirmed by colonoscopy, which revealed an ulcerated, bleeding large neoplasm distally to the hepatic flexure (transverse colon), almost causing total occlusion. Biopsy reported suspicion of a squamous type of LC origin. Oncological markers, such as carcinoembryonic antigen (CEA), α-fetoprotein (AFP), CA 19-9, and CA-125, were all within the range. Total body examination was negative for other localizations. After a multidisciplinary evaluation, considering good control of primary cancer, optimal physical status, and high risk of occlusion, a laparoscopic enlarged right hemicolectomy was performed. The postoperative course was uneventful. Histologic examination revealed a tumor with complete wall thickness infiltration, which appears extensively ulcerated, from poorly differentiated squamous carcinoma (G3), not keratinizing, with growth in large solid nests, often centered by central necrosis. The lesion was located over 5 cm from the colonic margin, while proximally ileal transection was over 30 cm distally from the lesion. Two of the 30 isolated lymph nodes were metastatic. The omental flap and resection margins were free from infiltration.

To evaluate these high-grade and poorly differentiated malignant changes further, we performed properly controlled routine immunohistochemical (IHC) stains for cytokeratin 7 (CK7), caudal type homeobox 2 (CDX2), and cytokeratin 20 (CK20) based not only on the age, gender, and past medical history of the patient but also on her recent clinical, radiologic, and operative findings. The malignant cells exhibited a strong positive immunoreactivity only for p40, while the tumor was negative for CDX2, CK20, and CK7. The features supported metastatic squamous carcinoma of lung origin (S-NSCLC), rather than primary colorectal adenocarcinoma. This hypothesis was supported by numerous colonic and regional lymph node samples lacking malignant carcinoma cells and properly controlled IHC stains of the right colon and ileum biopsy cells exhibiting negative immunoreactivity for CK7 and TTF-1. In addition, PD-L1 was determined on this specimen, with an expression of <1%, while EGFR resulted without mutations (wild type).

**Figure 1:**
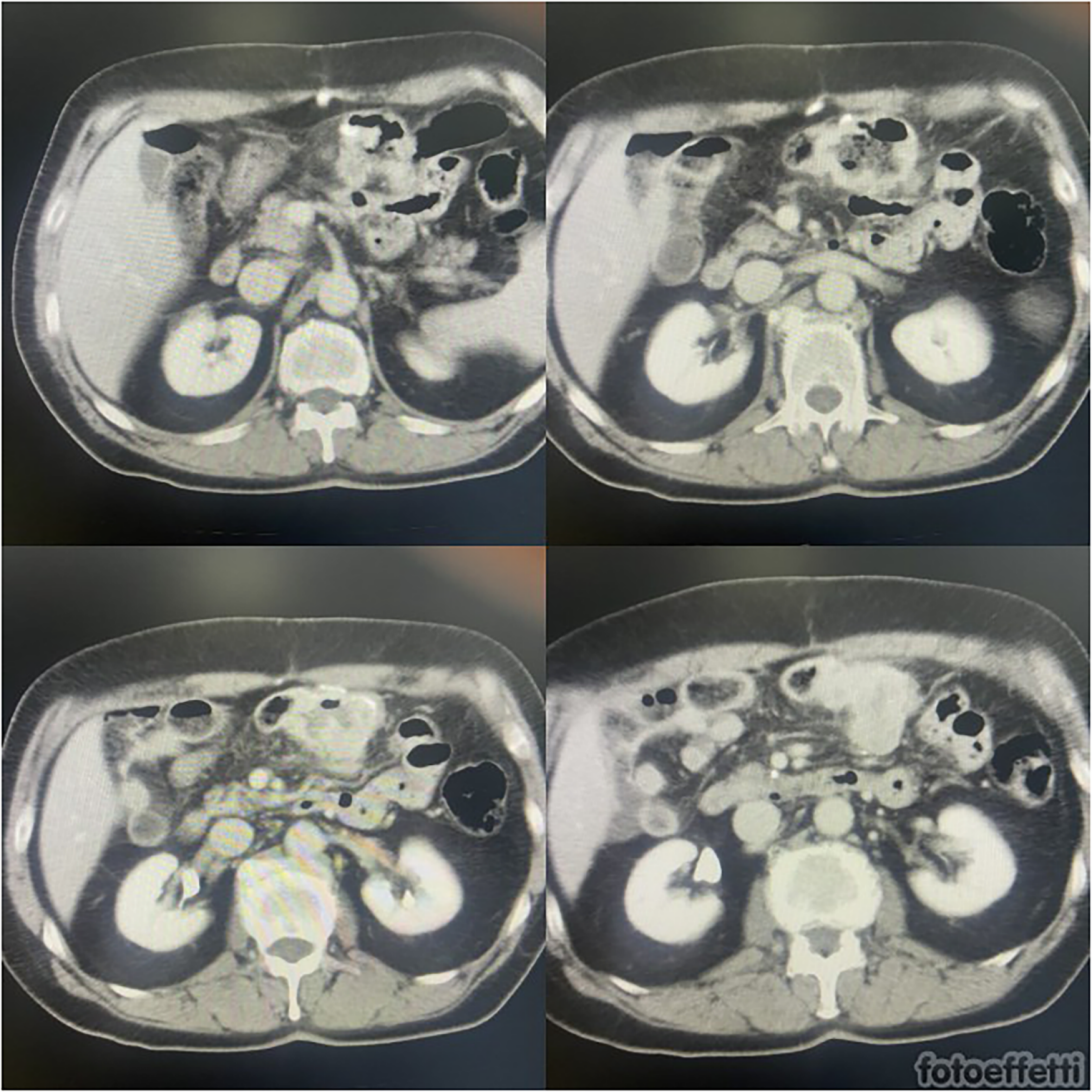
CT assessment of colon metastasis detected during follow-up.

After surgery, radiotherapy was associated with pembrolizumab to reinforce the effectiveness of treatment on primary cancer. After 8 months from surgery (26 months from diagnosis of S-NSCLC), subsequent PET-CT scans suggested substantial metabolic stability of the known right hilar lung lesion (SUV max: 6.6) and subcarinal lymphadenopathy (SUV max: 5.1) at the first follow-up. In addition, intense FDG uptake of tissue was confirmed in the transverse colon (SUV max: 38.3 vs. 35.6). It was suggestive of local relapse or peritoneal carcinosis. Abdominal magnetic resonance (MR) excluded peritoneal involvement, while colonoscopy evidenced an ulcerated substenotic area that involved ileocolic anastomosis on both sides. The multidisciplinary evaluation confirmed surgical indication, considering stable local primitive cancer, good general condition, and a high risk of complications (Figure 1).

Reoperation consisted of radical resection of ileocolic anastomosis with local lymphadenectomy and ileotransverse anastomosis 5 cm proximally and distally from the previous localization. The procedure required splenic flexure preparation. Intraoperative exploration excluded posterior infiltration of the gastric and duodenal walls.

No surgical complications were observed, and the patient was discharged on the sixth postoperative day (POD). On the third POD, asthenia, fever, and general hypomotility occurred. Due to the chronicle therapy with prednisone (25 mg/die), we hypothesized postoperative adrenal insufficiency by the abolition of the hypothalamus–pituitary axis. Starting with an endovenous therapy with hydrocortisone (bolus of 500 mg and subsequent therapy with 200 mg every 12 h), the symptoms were solved in 24 h, confirming the diagnosis. The endocrinological evaluation indicated chronic treatment with hydrocortisone per os, until recovery of the hypothalamus–pituitary axis.

The second histologic examination also revealed a tumor with full wall thickness infiltration of ileocolic anastomosis, which appears extensively ulcerated, from poorly differentiated squamous carcinoma (G3), not keratinizing, with growth in large solid nests. Neoplastic perineural infiltration and embolus were present. The lesion was located over 4 cm from the colonic margin on both sides. One of the isolated lymph nodes was metastatic. The omental flap and resection margins were free from infiltration. On routine IHC, the malignant cells exhibited strong positive immunoreactivity only for p40, while the tumor was negative for CDX2, CK20, and CK7 (confirming S-NSCLC metastasis). The features supported metastatic squamous carcinoma of lung origin (S-NSCLC). Similar genetic results were found also on this specimen (PD-L1 <1% and EGFR wild type).

After surgery, the patient started chemotherapy platinum-based for advanced LC. At 18-month follow-up, the patient was free from disease at PET-CT evaluation and asymptomatic. He continued immunotherapy with a standardized schedule and finished steroid therapy.

## Discussion

We report a peculiar case of colonic metastasis from LC that relapsed after 8 months at the site of anastomosis after colonic resection of metastasis, although radical resection was performed. Its uniqueness is related to the same location of metastasis, related probably to lymphovascular involvement of this gastrointestinal site.

The most common metastatic sites of LC are bone and brain (over 30%), but many other locations were discovered during the evolution of this cancer (2). The incidence rate of gastrointestinal metastasis of primary LC ranges from 0.3% to 1.7% (4). Interestingly, the incidence ranges between 4.6% and 14% in postmortem studies (5). This discrepancy indicates that most patients have asymptomatic gastrointestinal metastases. LC metastasis can spread to any gastrointestinal location from the oral cavity to the anus through lymphatic and hematogenous pathways being the probable routes of spread (4, 5). Specifically, colonic metastasis is uncommon with an incidence rate of 0.1%: it has been reported by numerous studies, but the incidence in autoptic series is relevant (4, 6). Kim et al. (7) reported 10 (0.19%) out of 5,239 patients with LC metastasis to the colon and rectum. A literature review of 15 cases of metastatic LC to the colon demonstrated that S-NSCLC is the most common subtype, similar to that in our report, while adenocarcinoma had the second highest potential for colonic metastasis (8). Approximately 50 case reports of LC metastasizing to the colon have been published worldwide, with various cellular differentiations.

The majority of cases of this disease are diagnosed in symptomatic patients, and detection during follow-up, similar to that in our case, is uncommon (6). With over 12% of colonic metastasis from LC in autoptic series, detection during follow-up is also uncommon (9). Gastrointestinal complications usually occur after the diagnosis of LC is established (6). Sometimes they can occur early in the course of the disease or even before an LC diagnosis has been made. Metastatic LC to the colon was also associated more with serious complications such as perforation, hemorrhage, and intussusception (6, 9).

Synchronous colonic metastasis is rarely described (10). Initial diagnosis of colonic metastasis of LC is challenging since its incidence has been reported sporadically. This phenomenon has been reported more frequently due to the recent higher rates of long-term survival of LC patients, increased availability and utilization of endoscopic examinations, and advancements in immunotherapy (10). Differently from primary cancer, an asymptomatic diagnosis of colorectal metastasis can be associated with a worse prognosis (10). This is related to the oncological evolution of primary neoplasm. Moreover, early detection and surgical intervention have been postulated to improve survival (11).

Moreover, it is very difficult to distinguish if the colon is the primary cancer site or a metastasis from LC. Despite that, CT scans can miss small asymptomatic gastrointestinal lesions that can be detected with PET-CT scans (12). Specimen histology is useful for suspecting the pulmonary origin of colonic lesions, as evidenced by many case reports. Endoscopic and clinical data are not specific for this differentiation, but a previous endoscopic biopsy is very useful for suspicion of colonic metastasis (6). Histological examination on intestinal resection, in correlation with the clinical findings, remains the gold standard for diagnosis (12). IHC is of utmost importance for the diagnosis and differentiation of metastasis from primary colonic malignancy (13). Colorectal adenocarcinoma is typically CDX-2 positive, cytokeratin (CK) 20 positive, and CK7 negative (13). Most primary lung malignancies are TTF-1 and p40 positive and CK7 and CK20 negative (14).

The average survivability of patients with LC varies widely, from the time of diagnosis of colonic metastasis to death. Moreover, outcome data for small and large bowel metastases are often aggregated. The 5-year survival rate for stage IV metastatic NSCLC is approximately 10% (2, 4). All forms of intestinal metastasis of LC are considered late-stage complications of the disease. However, a new concept of *oligometastatic* disease was introduced just over two decades ago and has been expanded to a multitude of cancer types, such as NSCLC (2, 15). Notably, oligometastatic NSCLC may represent a disease state with limited disease burden amenable to localized therapy (i.e., resection, radiation, and ablation) and improved survival outcomes similar to that of locoregionally advanced NSCLC (16). A recent single-institution retrospective review showed that patients with both synchronous and metachronous oligometastases from NSCLC had excellent overall survival (OS) (median OS = 21.8 months) when the oligometastatic disease was treated radically either with surgery or stereotactic radiation (17). Lengthy OS has been shown in patients with oligometastatic LC to a variety of organ sites (16). This approach reinforced the surgical indication of our case, either in the first recurrence or the second colonic recurrence. This new concept also includes new oncological targeted drugs. The combination of chemotherapy and platinum-based pemetrexed and carboplatin is the first-line treatment for advanced NSCLC. Our patient initially received pembrolizumab before the discovery of colonic metastasis. This is a novel and well-researched cancer immunotherapy most commonly used for tumors that are unresectable, recurrent, or metastatic (18). Until recently, pembrolizumab has been recommended as a second-line agent (19). Trends are now focusing on tumor genotype-specific characteristics and in favor of earlier use of immunotherapeutic agents (20). These are generally associated with fewer adverse events compared to platinum-based chemotherapy.

Regarding the therapeutic approach to colonic metastasis, the goal is the choice of surgery. In the case of synchronous metastasis, the most important decision is which lesion needs to be treated first. It depends on the extent of colonic metastasis and the nature of the presentation. For patients with complicated colonic metastasis, proper surgical treatment provides better outcomes in terms of complication rate, quality of life, and palliation (5). In the case of metachronous metastasis, the risk of complications (bleeding, obstruction, perforation) and evaluation of life expectancy from LC are two topics in the surgical approach (6, 21). When a primary disease is controlled and other locations are lacking, similar to that in our report, surgery is mandatory and safe (22). This is valid properly also when a second location is detected. As evidenced in the description of the case, the same location of the second metastasis is an unfortunate condition that may be related to local massive lymph node involvement or micrometastasis located at other sites of the transverse colon. Moreover, an omental flap free from disease at the first procedure is another interesting question: relapse was observed, despite clear peritoneal invasion. This infrequent and atypical oncological course will need other reports for assessment. We hypothesized that lymphnodal diffusion and probably combs of cells in microcircle led to early recurrence, similar to primary cancer.

Our case is unusual and peculiar for almost three characteristics: asymptomatic diagnosis in both detections; early second recurrence at the site of anastomosis, although radical surgery, confirmed by pathological specimen; and no systemic or peritoneal disease on both histological studies. In view of the second point, another similar case was described, reporting a large tumor at the colonic anastomosis with a specimen consistent with primary NSCLC (23). Moreover, in this case, the previous intestinal resection was for primary colonic cancer, while the first resection was for metastatic LC in our report.

## Conclusion

Colonic metastasis should be considered when patients have abdominal symptoms and a history of primary LC. In literature, like our report, PET-CT scans and endoscopy are highly sensitive for detection, but histological examination with IHC confirms the diagnosis. This case report confirmed that in asymptomatic patients and those with a history of LC diagnosis, the possibility of colonic secondary disease should be part of the differential diagnosis. In addition, relapse of colonic metastasis is infrequent but should be considered during follow-up of LC. Surgical treatment, associated with medical therapy, is useful, in consideration of reasonable life expectancy, good control of LC, and high risk of complications. More studies on colonic metastasis of LC are required to better understand the clinical features and outcomes.

## Data Availability

The original contributions presented in the study are included in the article/Supplementary Material, further inquiries can be directed to the corresponding author.
